# From Basic Mechanisms to Clinical Research: Photodynamic Therapy Applications in Head and Neck Malignancies and Vascular Anomalies

**DOI:** 10.3390/jcm10194404

**Published:** 2021-09-26

**Authors:** Daniele Ramsay, Harvey Stevenson, Waseem Jerjes

**Affiliations:** 1 Imperial College School of Medicine, London SW7 2DD, UK; daniele.ramsay18@imperial.ac.uk (D.R.); harvey.stevenson18@imperial.ac.uk (H.S.); 2 North End Medical Centre, 160 North End Rd, London W14 9PR, UK; 3 UCL Department of Surgery, University College London Medical School, London WC1E 6DE, UK

**Keywords:** PDT, nasopharyngeal cancer, oral cancer, oropharyngeal cancer, laryngeal cancer, vascular anomalies, head and neck

## Abstract

Head and neck cancers are largely squamous cell carcinomas derived from the epithelial lining of the structures in the region, and are often classified anatomically into oral, oropharyngeal, nasopharyngeal and laryngeal carcinomas. The region’s component structures serve complex and intricate functions, such as speaking, swallowing and breathing, which are often compromised by these neoplasms. Such lesions may also cause disfigurement, leading to distressing social and psychological issues. Conventional treatments of these neoplasms usually involve surgical intervention with or without chemoradiotherapy. These have shown to be efficacious; however, they can also cause damage to healthy as well as diseased tissue, exacerbating the aforementioned problems. Access to a given region to deliver the treatments is also often a problem, due to the complex anatomical structures involved. The use of photodynamic therapy in the head and neck region has been established for about two decades. In this review, we looked at the basic mechanisms of this intervention, examined its use in common head and neck malignancies and vascular anomalies, and reported on the most recent clinical studies. We further included a clinical guide which can help replicate the use of this technology by any unit. Based on this review, photodynamic therapy has been shown to be efficacious in the treatment of head and neck malignancies and vascular tumours. This therapy can be targeted to the diseased tissue and causes no damage to underlying structures. Recent studies have shown this therapy to be as effective as conventional therapies, without causing major adverse effects.

## 1. Introduction

Photodynamic therapy (PDT) is a minimally invasive interventional modality used in the treatment of various tumours in various anatomical locations. Fundamentally, PDT relies on three components: a photosensitiser (chemotherapeutic substance), light (laser), and oxygen. Initially, a photosensitiser is administered either systemically or topically, and specific wavelength light is then applied with a delay of several hours (drug–light intervals). The delay allows the photosensitiser to localise in the pathological tissue, which in basic terms is believed to be due to the increased vascular permeability of malformed blood vessels and a lack of tumour lymphatic drainage. The effectiveness of PDT is affected by a few factors: the concentration of the photosensitiser in the pathological tissue, the light intensity and dose rate, oxygen availability, and the localisation of the photosensitiser within the cell. The resultant reaction from the three components leads to the ischemic and direct tumour cell death (apoptosis) of the pathological tissue. Exploring various dosing regimens, along with the development of new and potentially oxygen-independent, conjugated photosensitisers, will lead to improvements in treatment outcomes and a wider application of PDT [1,2,3].

PDT has several benefits over traditional treatment modalities (i.e., surgery, chemoradiation). Photosensitisers have intrinsically low systemic toxicity as they require light activation; this, in conjunction with the non-thermal nature of the photochemical reaction, reduces the rates of PDT-associated morbidity and disfigurement when compared to conventional treatments (preservation cellular collagen). An important drawback is the long period of photosensitisation post-treatment; however, photosensitivity reactions can be avoided with small and gradual increases in light exposure [4,5].

### 1.1. Head and Neck and PDT

The use of PDT in the treatment of head and neck pathology has been steadily advancing in recent years, as its use has been evaluated and its role is now being elucidated. This modality has been used for curative or palliative intent depending on the pathology’s size, nodal involvement and distant metastasis.

For example, PDT was used in the treatment of early-stage head and neck squamous cell carcinoma (HNSCC) in three studies, which found it showed high levels of effectiveness, with 72.7% and 69.0% of tumours recorded from one centre, and 69.0% of tumours from another centre showing complete response (complete and partial responses at 97.6%) [6,7,8]. These results are encouraging, as a greater degree of preservation of function was noted when compared to traditional therapies.

PDT has also showed promise in the palliation and treatment of advanced HNSCC, with substantial symptomatic relief and improvement of function observed. In a study conducted by Lambert et al., 26 patients with oral SCC (OSCC) or oro-pharyngeal SCC (OPSCC) underwent PDT. A complete response was seen in 76.9% of cases post-PDT. Subsequently, recurrence occurred in nearly two thirds of the patients (*n* = 21), with a median survival of 24 months recorded, suggesting PDT could play a role in the treatment of advanced and/or recurrent disease. Another study of 128 patients with advanced disease recorded a complete response in 38% of tumours, with 58% of tumours undergoing a >50% tumour mass reduction. The study also reported a clinical quality of life improvement (QoL) in 61% of patients, indicating the potential role of PDT in alleviating tumour-related symptoms [9].

Potential issues associated with PDT treatment include pain and swelling of the treatment area. Due to the anatomy of the head and neck, this can lead to a temporary reduction in function resulting from compression of the larynx and other structures. Pre-emptive measures have to be employed to reduce risks to visual, breathing, swallowing or speech problems. In order to mitigate these short-term complications of PDT, pain medication, steroid use, and airway support (either in the form of a nasopharyngeal tube or short-term tracheostomy) are some of the few measures that can be employed [10].

In this review, we looked at the basic mechanisms of this intervention, examined its use in common head and neck malignancies and vascular anomalies, and reported on the most recent clinical studies. We further included a clinical guide which can help replicate the use of this technology by any unit.

### 1.2. Theoretical Principles of PDT

**Figure 1:**
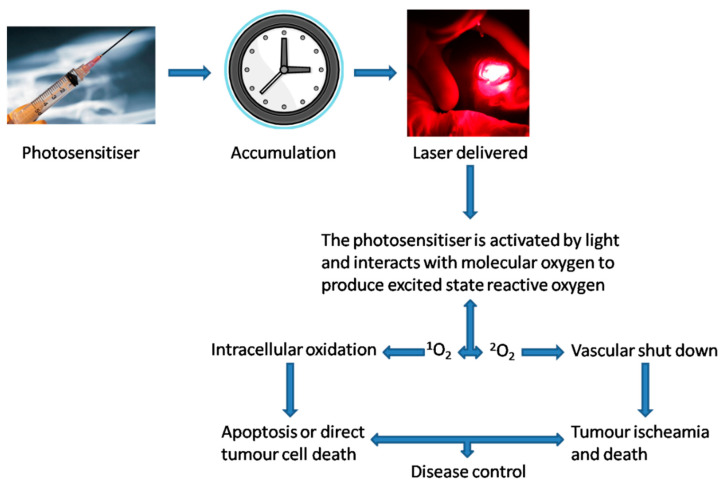
Theory behind the stages of photodynamic therapy application. Reprinted with permission from ref. [10]. Copyright 2010 Clin. Oncol. (R. Coll. Radiol.).

PDT relies on three main components: a photosensitiser, oxygen and visible light. Photosensitisers are usually administered systemically (slow intravenous infusion) but can be applied topically when managing certain superficial skin or oral pathologies. The photosensitiser is given time to accumulate in the target tissue before light of a specific wavelength is directed onto the lesion site: the drug-light interval (DLI) (Figure 1) [11].

When the pathological tissue is illuminated by the laser light, the photosensitiser absorbs a photon and enters its excited singlet state. Here, an electron is shifted to a higher-energy orbital, after which it returns to its ground-state, releasing the energy as heat or fluorescence. This phenomenon is useful for diagnostic purposes. Concurrently, a population of the photosensitiser will enter an excited triplet state, in which it can transfer its energy by phosphorescence or by colliding with other molecules. These collisions create chemically reactive species.

In competition with phosphorescence, a triplet state can either undergo electron transfer with organic substrates to create radical species (type I reaction) or directly react with ground-state oxygen to form singlet oxygen, a highly reactive oxygen species (ROS) (type II reaction) [10,11].

These resulting species are cytotoxic, due to their ability to react with biomolecules. Most of the cytotoxic effect is thought to be achieved by the production of ROS. Given the maximum action radius of singlet oxygen is about 20 nm, PDT is theoretically a highly specific and controllable therapy, with minimal systemic involvement [12].

PDT is thought to initiate both the necrotic and apoptotic sequences, depending on the site of photosensitiser activation. Photosensitisers which accumulate near the mitochondria tend to cause permeabilisation of the mitochondrial membrane, causing the release of cytochrome c and the subsequent activation of the caspase-mediated apoptotic pathway. However, in higher doses of PDT, components of the apoptotic pathway may be damaged, resulting in necrosis. Necrosis also tends to predominate when the photosensitiser targets the plasma membrane, which can be achieved by altering the DLI. Activation of the necrotic sequence results in loss of cellular contents into the interstitium, leading to an inflammatory response [13,14,15].

PDT is taken up by both healthy and diseased cells. However, solid tumours often have disorganised vascular supplies with defective inner endothelial linings, increasing the permeability and therefore the amount of photosensitiser that extravasates. Coupled with poor lymphatic drainage, which leads to longer retention, the concentration of photosensitiser in diseased tissues is higher than in healthy tissues [16].

While excitation of the photosensitisers within cells leads to cell death, targeting the tumour vasculature (i.e., intimal hyperplasia) by altering the DLI can lead to the same effect, by starving the existing tumour cells of oxygen and preventing further angiogenesis, which is detrimental to further proliferation. This is followed by the initiation of an inflammatory/immune response targeting the residual pathological tissue [11,15].

As PDT is a non-thermal photochemical process, there is no tissue heating, and connective tissue structures such as collagen and elastin are largely unaffected. There is therefore much less risk to the integrity of underlying structures than with thermal laser techniques and surgery [17,18,19].

PDT light sources include light-emitting diodes and xenon lamps, which are commonly used for dermatological applications, but lasers are the most convenient and controllable light source. Laser light is coherent and monochromatic and can be directed along fibre-optic cables, allowing light to be introduced into hollow organs and deep-seated tumours. Diode lasers, which are much cheaper and more portable than metal vapour or tuned-dye lasers, have become the preferred light source. However, non-laser light is preferred for dermatological applications, due to its larger field, low cost and simple construction [19,20,21,22].

### 1.3. Photosensitisers

#### 1.3.1. First-Generation

Porfimer sodium (Photofrin^®^ Concordia Laboratories Inc., Pinnacle Biologics, Bannockburn, IL, USA) was the first photosensitiser to receive approval, and it is now licensed for use in the oesophagus, lung, stomach, cervix and bladder. The peak of the absorption spectrum for porfimer sodium is 405 nm, but light of this wavelength penetrates biological tissues very poorly. Whilst it can be excited by light with a wavelength of 630 nm, it is only moderately active, as this wavelength still penetrates tissue only slightly, and the absorption band at this wavelength is weak; therefore, the depth of effect is limited to 0.5 cm. The efficiency of the transfer of energy from light to cytotoxic products (quantum yield) is moderate (about 0.5), and skin photosensitivity persists for many weeks because of the high doses required, long half-life and high accumulation in the skin. Using this haematoporphyrin, a maximum absorption can be reached at 630 nm with a drug dose of 2 mg/kg and a DLI of 48–72 h, a fluence of 100–200 J/cm^2^ and a fluence rate of 100 mW/cm^2^ [22,23,24].

#### 1.3.2. Second-Generation

Second-generation synthetic photosensitisers have shorter periods of photosensitivity (due to faster elimination from the body), longer activation wavelengths and, therefore, increased depth of effect, higher yields of singlet oxygen and better tumour selectivity. The groups that have been most actively investigated are chlorins, texaphyrins, purpurins, and phthalocyanines [24].

One example used here is 5-aminolevulinic acid (5-ALA), which is a naturally occurring precursor in the heme biosynthetic pathway, and to date it has received approval only for non-malignant disorders and actinic keratosis (AK). It is converted to the endogenous photosensitiser protoporphyrin IX, which can be activated by red, green and even blue light. Its uses are constrained by its depth of effect (<0.2 cm) to cutaneous lesions. It has been successfully applied in the management of basal cell carcinoma (BCC), actinic keratosis and oral premalignant disorder. With the formation of protoporphyrin IX, a maximum absorption can be reached at 635 nm. Since it is a prodrug, it can be administered topically in a 20% paste or systemically (oral 60 mg/kg or IV 30 mg/kg). The DLI is 3–6 h with a fluence of 100 J/cm^2^ and a fluence rate of 100–150 mW/cm^2^ [24].

Another example is Meso-tetrahydroxyphenyl chlorin (mTHPC-Foscan^®^ Biolitec Pharma Ltd., Jena, Germany), which is a more potent photosensitiser for cancer management compared to photofrin and 5-ALA, and is commonly used in advanced head and neck cancer. Maximum absorption is at 652 nm with a drug dose of 0.05–0.15 mg/kg, a DLI of 96 h, a fluence of 10–20 J/cm^2^ and a fluence rate of 100 m W/cm^2^ [21].

#### 1.3.3. Third-Generation

The main issue with second-generation photosensitisers is their poor solubility in water, limiting their use in intravenous applications. A good third-generation photosensitiser should be hydrophilic, which is better for clinical use [24,25]. Other characteristics of a third-generation photosensitiser should include optimal excitation wavelengths over 650 nm (to allow deeper penetration into tissues), and it should both be rapidly metabolised and eliminated from the body (for shorter generalised photosensitivity) and selectively accumulate in cancer cells [25].

#### 1.3.4. Specific Type

Disulfonated tetraphenyl chlorin (TPCS2a—Amphinex^®^ PCI Biotech, Oslo, Norway) is used to initiate the photochemical internalisation (PCI) process with a selected chemotherapeutic agent. The process can be best described as sub-lethal PDT that facilitates the effect of chemotherapy to reduce resistance and increase effectivity. TPCS2a is usually given approximately 93 h prior to a slow bleomycin infusion and subsequent illumination with a diode laser to initiate the PCI process [26].

### 1.4. Delivery of Photodynamic Therapy

**Figure 2:**
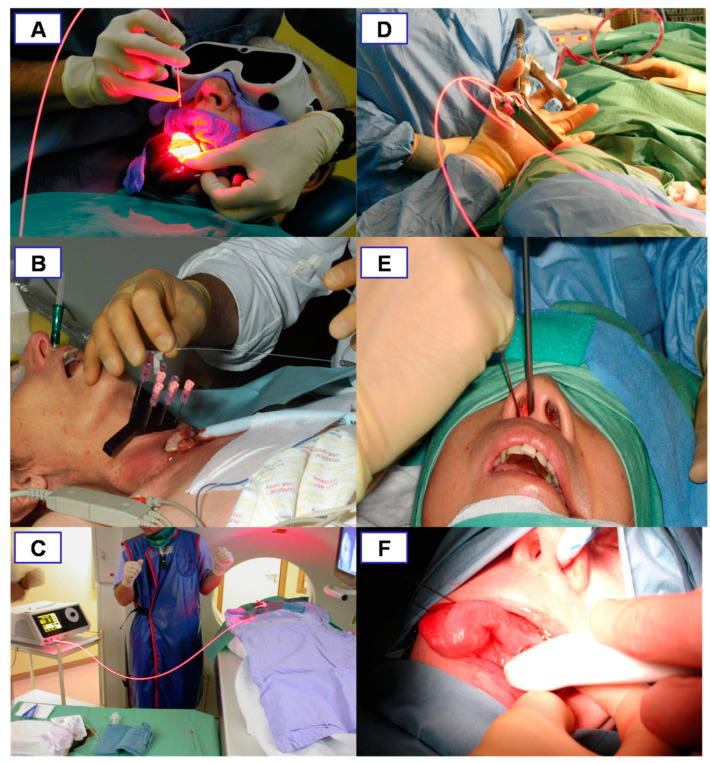
Mode of light delivery. (**A**): Superficial light application using a fibre-guided laser. (**B**): Interstitial photodynamic therapy using a pre-made grid to insert needles. (**C**): Interstitial photodynamic therapy with intraoperative MRI scanning. (**D**): Laryngoscopy employed to guide Photodynamic therapy(PDT). (**E**): Nasoendoscopy employed to guide PDT. (**F**): US employed to guide PDT. Reprinted with permission from ref. [10]. Copyright 2010 Clin. Oncol. (R. Coll. Radiol.)

The components involved in photodynamic therapy are a photosensitiser, light, and oxygen. Of these, both the light and photosensitiser need to be introduced to the tumour area in order to allow the formation of ROS, disruption of vasculature, and initiation of an immune response, which lead to the death of cancerous tissue and tumour size reduction [19]. PDT can be used for both superficial and deep-seated pathologies; however, there are some important differences between the two, which are discussed below. Surface pathology is generally more easily accessible, and when treating large superficial tumours, they can be debulked surgically prior to PDT treatment; PDT is then used to target the remaining tumour tissue. With deep-seated pathology, careful planning and insertion of fibres into the tumour area is required in order to target the tumour tissue effectively. In order to determine the accurate location, depth and overall size of the tumour, ultrasound (US), computed tomography (CT) and/or magnetic resonance imaging (MRI) are used in the planning stage pre-PDT delivery (Figure 2) [27]. These imaging modalities are often accompanied with endoscopic investigation of the area, allowing direct visualisation of pathology in difficult to reach anatomical locations (e.g., nasopharynx, oropharynx, larynx) [10]. During the procedure, intraoperative image guidance is used to guide the localisation of needles and fibre optic insertion, limiting light exposure to healthy tissue. Optical coherence tomography is also being investigated as a minimally invasive technique for assessing tumour vasculature and response to PDT [28,29,30].

#### 1.4.1. Superficial Pathology

When treating superficial pathology, the photosensitiser may be applied either topically or intravenously. In a study conducted by Jerjes et al., for oral SCCs, mTHPC was used at a dosage of 0.15 mg/kg and a drug–light interval of 48–96 h. Safety margins of around 5 mm around the lesion were also illuminated to ensure appropriate light coverage, with the remaining tissue covered to avoid the excessive exposure of healthy tissue. The distance from the laser fibre tip to the pathological area was 5 cm, with a 3 cm spot diameter, and 20 J/cm^2^ delivered. When using mTHPC, 652 nm lasers are widely accepted as optimal, as this is the wavelength that leads to maximal absorption. Drug–light intervals vary from 48 h up to 120 h; other factors including spot-light and energy delivery of light remain largely preserved across reported studies. Post-treatment, limited exposure to light is recommended, with light sensitivity remaining for up to 4 weeks [9,31,32].

#### 1.4.2. Deep-Seated Pathology

When a tumour is bulky and not accessible superficially, guidance using imaging techniques such as MRI and CT pre-treatment and US intraoperatively is employed to determine the ideal location of optical fibre insertion; this technique is termed interstitial photodynamic therapy (iPDT). Catheters are inserted into the area of the tumour, and laser fibres are passed through the catheters and into the tumour bulk itself. The area is then illuminated.

Once CT and/or MRI is carried out, the positioning of the fibres is decided under supervision of a senior clinician. Locations are drawn out at a distance of around 10–15 mm away from each other to account for the 10 mm penetration distance of 652 nm wavelength light in tissues. The area of necrosis around fibres has been reported to be 1 cm, which further justifies this placement distance. A safety margin of 5 mm into healthy tissue is also included in planning. A 1 cm safety margin of fibre placement away from critical structures such as the carotid arteries is recommended, and the use of intraoperative US guidance is imperative to ensure safety margins from vital structures. Grids can be made using the pre-planning imaging to ensure the ease and reliability of needle insertion [33,34,35,36,37].

The principal photosensitiser used for deep seated pathology is mTHPC, with a standard dose of 0.15 mg/kg used in most cases. The drug–light interval is generally accepted to be at 96 h to allow for localisation of the photosensitiser into the pathological tissue. During this time, patients are withheld from intense light; post-PDT patients are advised to avoid intense light sources for up to 4 weeks. Exposure during this period of prolonged photosensitisation may result in photosensitivity reactions such as skin burning and hyperpigmentation. The most common, immediate treatment-related post-PDT side effects have been widely reported as pain in the treatment site, oedema, and bleeding [33,34,35,36,37].

**Figure 3:**
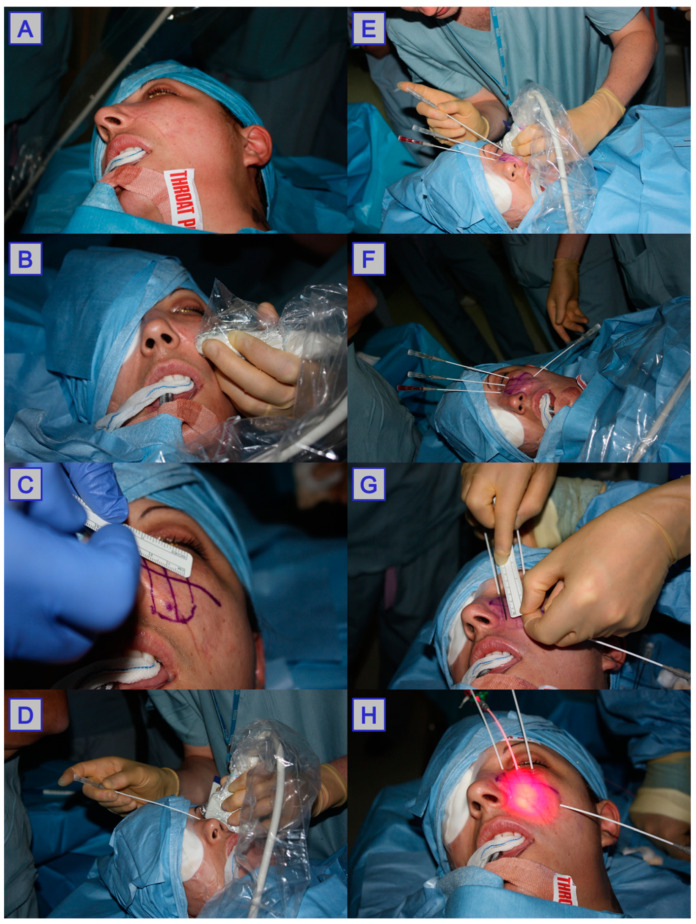
The steps involved in the interstitial PDT application to treat a left infraorbital haemangioma. (**A**): The patient is prepared with a drape and eye shield. (**B**): US is used to accurately identify the location of the lesion. (**C**): The locations for needle insertion are mapped out superficially. (**D**): US is used during the insertion of the first needle. (**E**): US is used for the insertion of subsequent needles. (**F**): The needles are inserted an appropriate distance from each other to ensure complete illumination of the tumour width and depth. (**G**): The needle tip in the tissue is measured to avoid damage in order to avoid necrosis of overlying healthy tissue. (**H**): The lesion is illuminated. Reprinted with permission from ref. [34]. Copyright 2011 Photodiagnosis Photodyn. Ther.

For light delivery, xenon lamps as well as light-emitting diodes have been used in the field of dermatology; however, specific wavelength lasers are more extensively used in iPDT for several reasons. Specific wavelengths can be used with 652 nm lasers used when employing mTHPC iPDT; other photosensitisers with different peak absorptions require different wavelength lasers. More specifically, four-channel 652 nm diode lasers with 400 µm core-diameter light-delivery fibres are most widely used; these can be passed through catheters flexibly to allow deeper tissue illumination. The diodes are moved out from the catheter or needle tip (18G) by 2–3 mm to allow for maximal illumination. The fluence rate and illumination times are set at a standardised 100 mW/cm^−1^ and 200 s, respectively. In order to illuminate the full thickness of the pathology, a pull-back technique is used [34,35,36,37] (Figure 3).

### 1.5. Post Photodynamic Therapy

#### 1.5.1. Pain Management and Swelling Control

**Figure 4:**
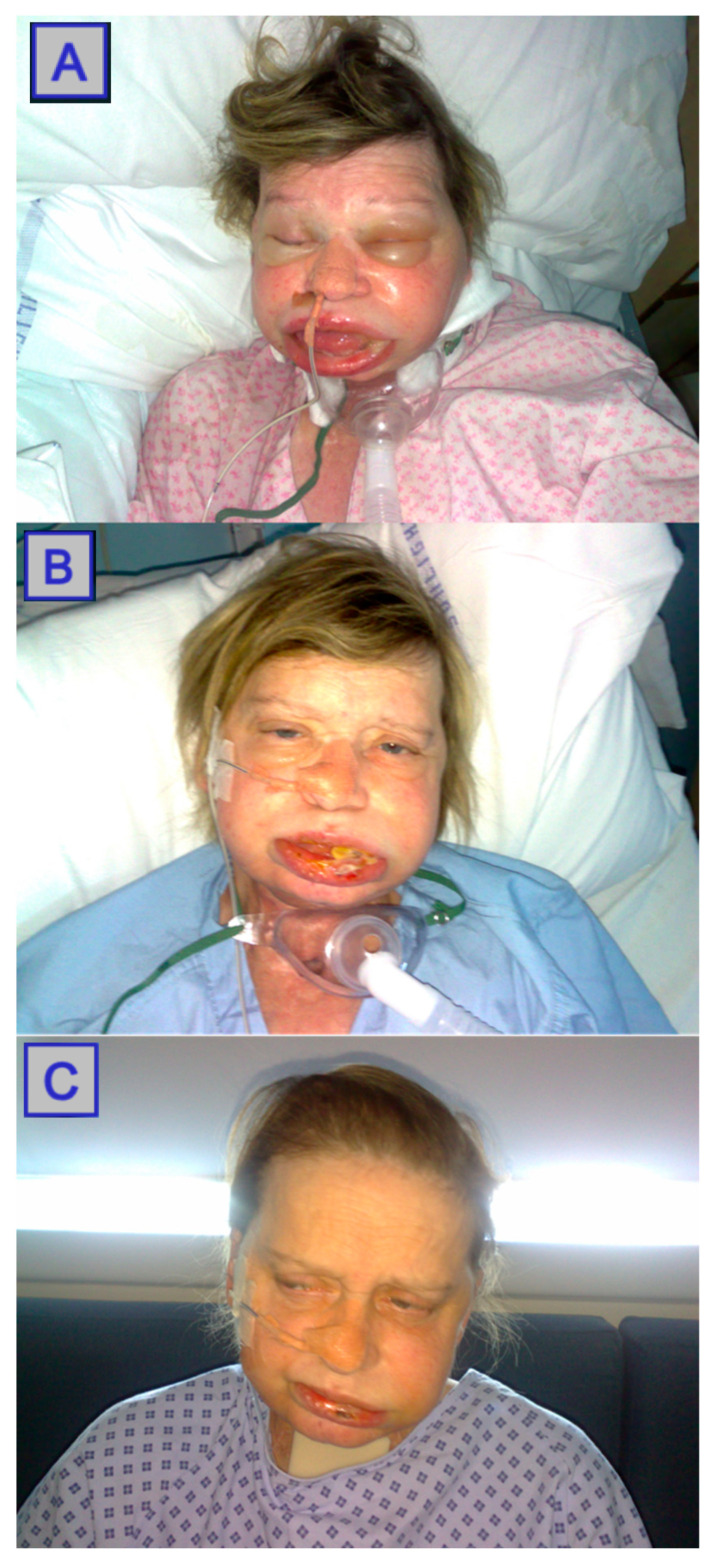
Images displaying post-PDT swelling. Day 2 post-PDT (**A**). Day 4 post-PDT (**B**). Day six post-PDT (**C**). Reprinted with permission from ref. [38]. Copyright 2008 Oncol. News.

Pain and swelling are the most common adverse events post-PDT. Pain is experienced by all patients post-PDT, and usually peaks at 48–72 h post-intervention. Special PDT pain protocols are followed, which usually differ centre-to-centre. NSAIDs and opiates are usually supplied if not contraindicated when managing superficial disease. At the end of the treatment, 10 mL of 0.5% bupivacaine is administered locally. No epinephrine is included, as this can cause vasoconstriction, hence compromising the efficacy of treatment [36], (Figure 4).

The standard regime, when managing a deep-seated disease in head and neck cancer patients, involves administering a 72 h fentanyl transdermal patch, with 12 mcg/h of morphine sulphate (immediate release) as needed for breakthrough pain. Dose-escalating the patient’s own pain medication or prescribing patient-controlled analgesics (PCA) is implemented when indicated [36].

Post-PDT inflammatory/immunology reactions can cause local swelling, leading to airway compromise. Airway control is, therefore, paramount. Elective tracheostomy prior to interstitial PDT is implemented when managing advanced tumours in the oropharyngeal/laryngeal region. For example, a tracheostomy tube can be inserted intra-operatively and kept for 3–5 days postoperatively to prevent airway compromise. Intravenous steroids (i.e., dexamethasone) are also administered for 3 days to reduce local tissue responses. The use of nasopharyngeal tubes has been found to be helpful in patients treated for oral pathologies using PDT [1].

#### 1.5.2. Residual Systemic Photosensitisation

The major side effect of PDT is residual systemic photosensitisation, which lasts for several days or weeks depending on the administered photosensitiser. This is caused by minor concentrations of the photosensitser in the skin, and may lead to oedema, sunburn, or even superficial skin necrosis when normal skin is exposed to bright light. The advantage of topically applied photosensitiser (i.e., 5-ALA) is the lack of systemic photosensitivity; therefore, patients do not have to avoid exposure to light following treatment. The major disadvantage of a topically applied photosensitiser is the small treatment depth of only 1–2 mm that can be obtained. Therefore, only very superficial lesions of less than 1 mm can be treated successfully.

The position of the pulse oximeter should be changed every 20–30 min to avoid any skin burn or nail bed damage that would result from photochemical reaction by the red light (660 nm). All precautions should be taken to avoid direct illumination of the patient with surgical lamps in theatres. Unplanned or emergency surgical interventions within 30 days from the photosensitiser administration should be undertaken only if absolutely necessary and if the potential benefits outweigh the risk to the patient.

**Figure 5:**
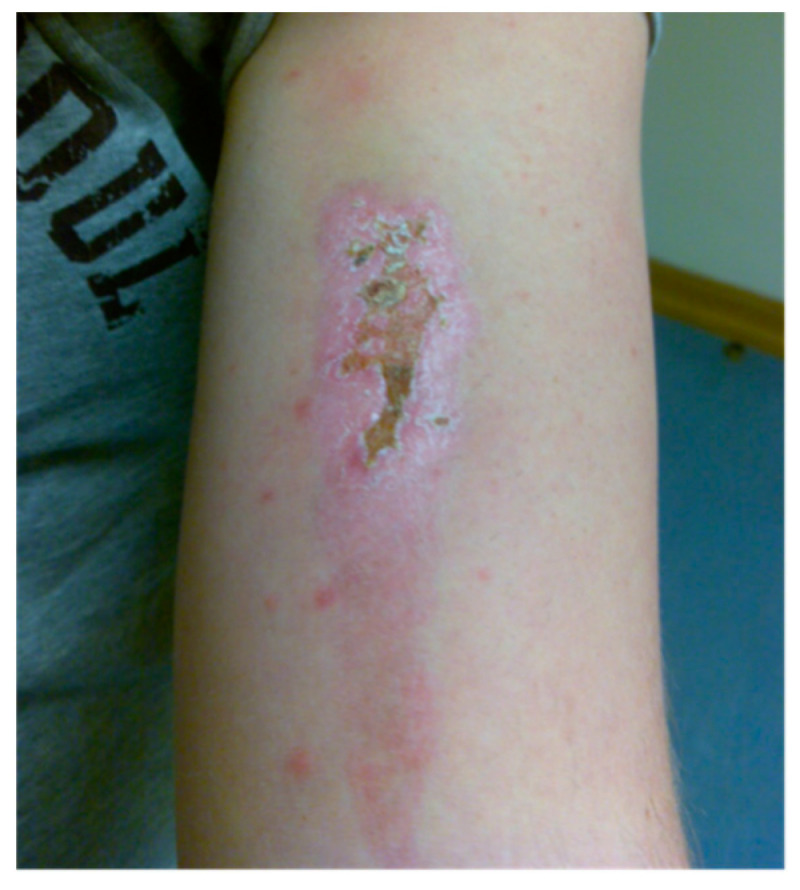
Image displaying the consequences of inadvertent sunlight exposure post-treatment. Patients are advised to follow post-treatment protocols when considering light exposure and other complications. Reprinted with permission from ref. [40]. Copyright 2009 Lasers Surg. Med.

Gradual light re-exposure at an incremental rate of 100 lux/day is usually implemented. Every patient is instructed on the need to avoid direct sun exposure for up to 2 weeks after injection (i.e., administration of the photosensitiser), and is given light exposure guidelines. Sometimes patients fail to achieve a gradual re-exposure to sunlight and as a result they develop skin burns, 1st or 2nd degree, when they are exposed for the first time to direct sunlight after 3–4 weeks of treatment. Additionally, the skin over the injection site is more sensitive to light, and skin burn has been reported to occur up to 10 weeks after the photosensitisation in this area [39], (Figure 5).

#### 1.5.3. Assessment of Outcome

Following PDT, most patients will take anywhere from 3 to 10 days to recover post-operatively, depending on the tumour location, side effects of treatment and comorbidities. As of yet, a QoL questionnaire dedicated to PDT used in head and neck oncology has not been developed, but a modified Washington questionnaire is commonly used and has been validated with other modalities. In most studies reported, QoL has been assessed in terms of general improvement of symptoms, but a more definitive metric is needed [39,41,42].

Several approaches are taken when assessing the extent of PDT effectiveness. When staging tumours, pre-operation CT or MRI is a standard procedure, and post-operation, the scans are repeated to quantify the response to treatment. The most commonly used guidelines for tumour response were initially the WHO criteria; however, more recently, assessment criteria such as RECIST have superseded the WHO criteria in clinical practice.

In one study conducted by Jerjes and colleagues, the radiological assessment of tumour bulk was conducted using a post-op MRI taken between 5 and 6 weeks post-PDT, with the parameters set as: no response indicating a lack of size reduction in tumour bulk; a minimal response, whereby tumour bulk reduced in size by <25%; a moderate response, with a size reduction of <50%; a significant response, indicated by a size reduction between 50% and 75% [42,43]. Another study conducted by the same team defined tumour response according to RECIST: a complete response defined as the lack of detectable tumour, a partial response, whereby tumour size reduced by >20%, and no response, indicating a reduction in tumour size by <20% [39].

RECIST criteria are the primary method for assessing anti-solid tumour efficacy; however, this “response to treatment” criterion is not without flaws. The main issue remains the difficulty in interpreting the criteria when assessing various tumours in various anatomical locations. Furthermore, the assessment of target and non-target lesions can vary with inter- and intra-observer variation.

Visual inspection of the area targeted with PDT is also undertaken to assess effectiveness. In a case series reported by Biel, multiple biopsies were taken at 1-month post-op to assess tumour response. An additional approach using fluorescence diagnosis to assess clinical response was used by Trunchuelo et al.; the team concluded that although the technique has not yet been validated, it could be used as an additional tool to assess response alongside histopathological biopsies [44,45].

### 1.6. Tissue Changes

PDT leads to several changes to the tissue environment. The formation of oxygen radicals in PDT leads to the consumption of oxygen in the tissue and, alongside the damage to local vasculature, leads to a significantly hypoxic area. These changes can be controlled to some extent by modifying the dosimetry of both PS and fluence [46].

Although there is a lack of detailed investigation in the literature, it is recognised that there are a few distinct phases treated tissue undergoes post-treatment, as described by Jerjes et al.

The initial acute inflammatory and immunological phase occurs almost immediately after treatment, with pain, oedema and an increase in inflammatory markers reported. In some treatment of superficial pathology, blistering can occur at this stage. This stage may last up to 3 days [47,48].

**Figure 6:**
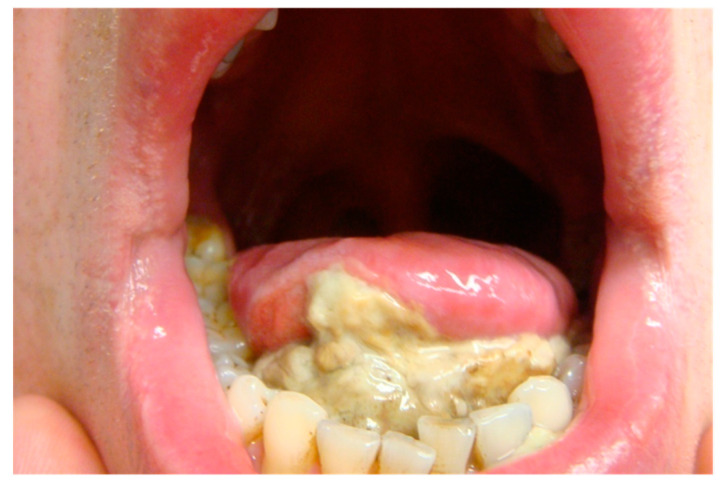
This image displays sloughing of necrotic tissue in the oral cavity post-PDT. Reprinted with permission from ref. [36]. Copyright 2011 Lasers Surg. Med.

**Figure 7:**
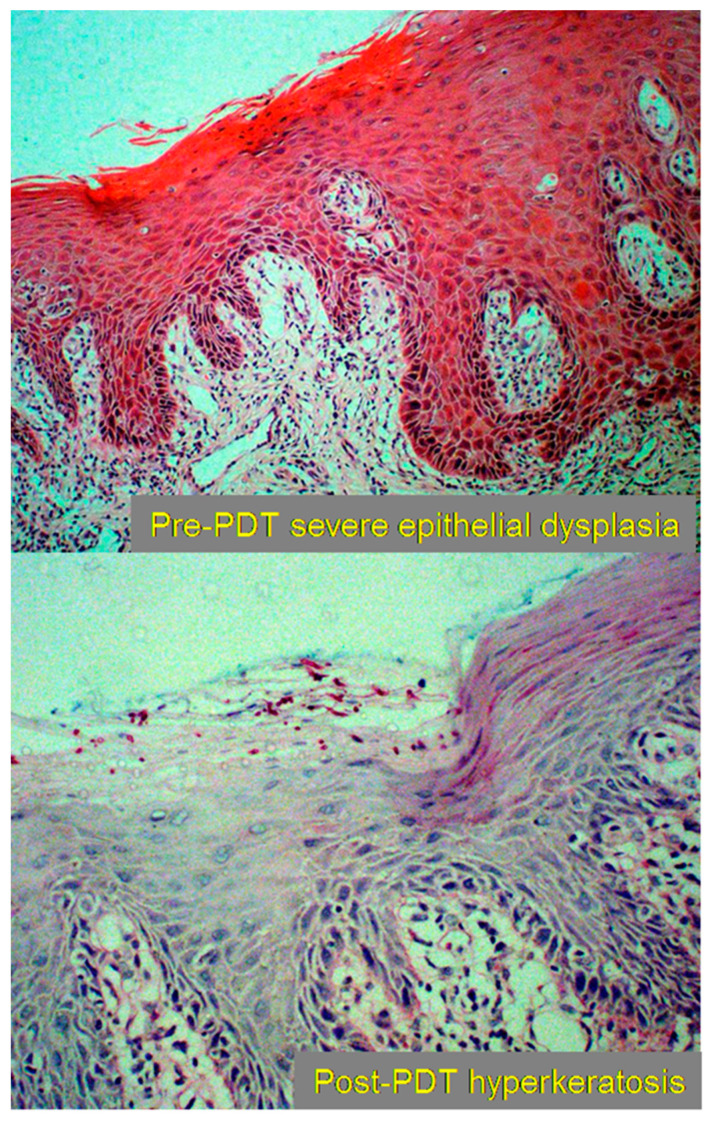
Histopathological slides displaying the replacement of dysplastic tissue pre-PDT with hyperkeratotic tissue post-PDT. Reprinted with permission from ref. [50]. Copyright 2011 Lasers Surg. Med.

The second stage involves apoptosis and necrosis of the tissue; this may occur in conjunction with the first stage, but can last up to 5 or 6 weeks depending on the treatment dosing and location. For superficial pathology, this may be identified as sloughing of necrotic tissue. For deeper pathology, areas of necrotic tissue can be identified by radiologists with experience in PDT [47]. The third stage involves the healing of the tissue, which begins from 2 weeks post-PDT, and can continue for up to 6 weeks post-PDT [47,49] (Figure 6 and Figure 7).

## 2. Discussion

### 2.1. Nasopharyngeal Carcinoma

Nasopharyngeal carcinoma (NPC) refers to malignancy arising from the epithelium of the nasopharynx. It is very rare, with a global incidence less than 1 per 100,000 person-years, and lower in most of the West. It is highly endemic in Southern China, with increased incidences in parts of South-East Asia, Northern Africa, and parts of the Middle East [51,52]. NPC susceptibility is thought to arise from an interplay between genetics and Epstein–Barr virus (EBV) exposure [53].

NPCs are highly radiosensitive, and radiotherapy is therefore the management of choice. It is effective for all cases except distant metastases [54]. NPCs are also highly chemosensitive; hence, in advanced regional disease, chemoradiotherapy is the mainstay of treatment, while chemotherapy is the management of choice when distant metastases are involved [55]. The nasopharynx is a small and deep region, rendering surgical access difficult. Therefore, surgery is reserved as a salvage treatment when encountering locally recurrent or residual NPCs [56].

The symptoms produced by NPCs vary depending on the position of the tumour in the nasopharynx, including nasal symptoms (such as obstruction, discharge with or without blood, post-nasal drip and vocal changes), otological symptoms (such as conductive hearing loss, effusions and fullness secondary to eustachian tube blockage), neurological symptoms due to cranial nerve involvement, and enlarged neck nodes in lymph node involvement [51]. These are obviously distressing for patients, and for advanced NPCs, conventional therapies often fail to control the symptoms until death [57].

**Figure 8:**
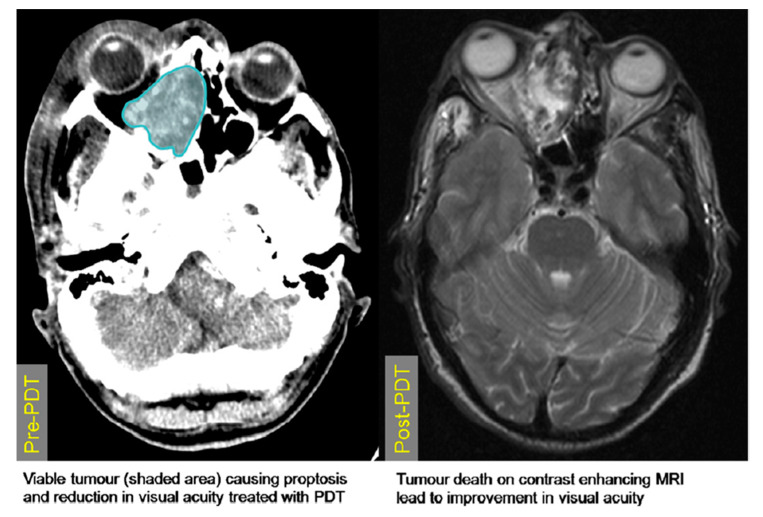
CT scans pre- and post-PDT of a mass located in the sinonasal area and extending into the retrobulbar space, affecting vision and causing proptosis. The post-PDT CT displays a reduction in bulk of the mass, leading to an improvement in visual symptoms. Reprinted with permission from ref. [43]. Copyright 2010 Photodiagnosis Photodyn. Ther.

**Figure 9:**
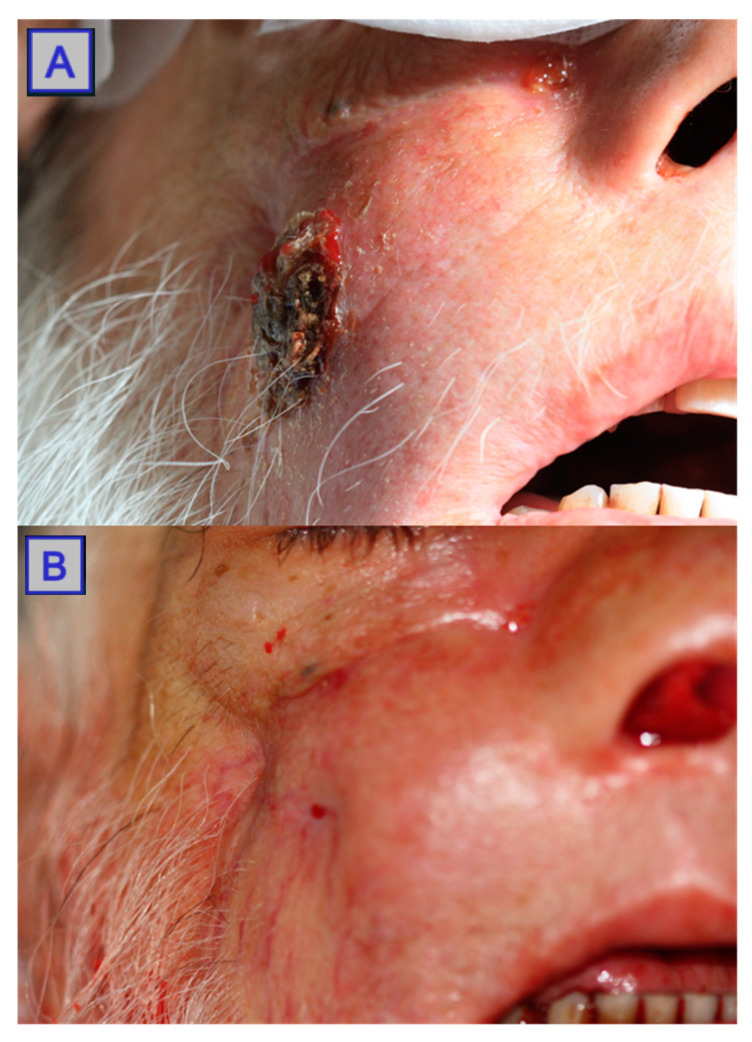
Images of a fungating tumour mass. Interstitial PDT was carried out on the mass (**A**). Post-PDT (**B**) shows a reduction in tumour size with no ulceration and extensive skin regeneration. Reprinted with permission from ref. [43]. Copyright 2010 Photodiagnosis Photodyn. Ther.

PDT plays a role where conventional therapies have failed to control the disease, or as a non-invasive alternative to salvage surgery (with its high morbidity) (Table 1) (Figure 8 and Figure 9). The largest case series was reported by Sun in 1992, where 137 NPC patients were treated with hematoporphyrin-derivative-PDT. Despite the age of this study, its size and impressive results (complete response achieved in 76 patients, marked response in 47) warrant mention in this review [57].

Since Yow et al. showed that mTHPC potentiated a 100-fold higher cytotoxic effect than hematoporphyrin derivates on nasopharyngeal carcinoma cell lines, subsequent studies have used mTHPC, which is licensed in Europe for use in advanced head and neck cancers [11,61].

Abbas et al. performed interstitial PDT on 7 patients with recurrent NPC. MRIs were taken at 6-weeks post-PDT, and radiological assessments of the response were made. One patient showed a minimal response (<25% decrease in tumour size), three showed a moderate response (25–50% reduction), and three showed significant responses to PDT (50–75% reduction). Five patients then had to undergo a second round, with one minimal, one moderate and three significant responses after the second round by radiological criteria. A 36-month follow-up revealed five mortalities (three of which were tumour-related and two of which were due to multiple-organ failure) [39].

Nyst et al. published their case series of 22 patients who were given m-THPC-PDT. The recommended dose was given alongside two regimens employing lower doses, which is used to evaluate whether it is possible to reduce the side effects and improve cost-effectiveness whilst maintaining efficacy. A total of 17 of the 22 patients were biopsied at 40 weeks: all showed no tumours. At a mean 58 months of follow-up, 10 had survived and 10 died (6 disease-related, 1 treatment-related, 1 unknown cause, 2 not related to disease or treatment), with 2 lost to follow-up. While the group sizes were very small, no significant difference in toxicity between the groups was noted, with the recommended dose group having better overall and disease-specific survival [58].

Succo et al. took six patients with recurrent or persistent NPC and administered PDT with palliative intent. For three of the patients, there was no suitable treatment, and for the other three, the conventional treatment was declined. At the time of the last follow-up (24–71 months post-PDT), three were disease free, one was alive with the disease, and two had died from disease [59].

Stoker et al. performed a phase II trial in 2015 evaluating the use of salvage PDT. A total of 20 out of 21 showed no tumours on biopsy at 8–12 weeks post-PDT. Overall survival at 2 years was 65%, with 9 (43%) being disease-free at the end of follow-up. No significant adverse effects were noted in any patient [60].

### 2.2. Oral Cancer

Oral squamous cell carcinoma (OSCC) is among the most commonly diagnosed neoplasia of the head and neck, and account for 90% of all oral cancers and between 2% and 4% of all cancer cases. OSCCs can occur throughout the oral cavity, but are more common on the tongue and floor of the mouth. When invading local structures, OSCCs can rapidly lead to disfigurement and loss of function. Their prevalence is greater in countries such as India and Pakistan; however, their rates of incidence are increasing worldwide, especially among younger patients. Risk factors include smoking and alcohol consumption in the West, along with betel consumption elsewhere; other factors represent a smaller risk for OSCC development [62].

Surgical excision is the mainstay of treatment, with radiotherapy used as a treatment option post-surgery in combination with chemotherapy for advanced disease. Despite advances in the treatments available, OSCCs have a 5-year survival rate of around 50%. One of the main reasons for this is the late stage at diagnosis, with misdiagnosis a potential reason for this, as well as the painless nature of the disease at early stages. Additionally, OSCCs may be difficult to differentiate from premalignant stages such as leukoplakia and erythroplakia, which all develop from oral keratinocytes [63]. Although treatments may be curative, they also present a range of quality-of-life (QoL)-impacting complications including xerostomia and cosmetic and functional defects [64]. For example, OSCCs commonly metastasise to the regional lymph nodes of the neck; therefore, neck dissection is carried out, which commonly results in dysfunction of the spinal accessory nerve [65].

Due to the poor prognosis associated with OSCCs, and the large impact on head and neck function caused by both the OSCCs themselves and treatment, alternatives and additives to conventional treatment are needed. Photodynamic therapy (PDT) has the potential to provide an additional tool to combat OSCC and to improve QoL in patients with advanced disease who are undergoing palliative treatment [66] (Table 2).

**Figure 10:**
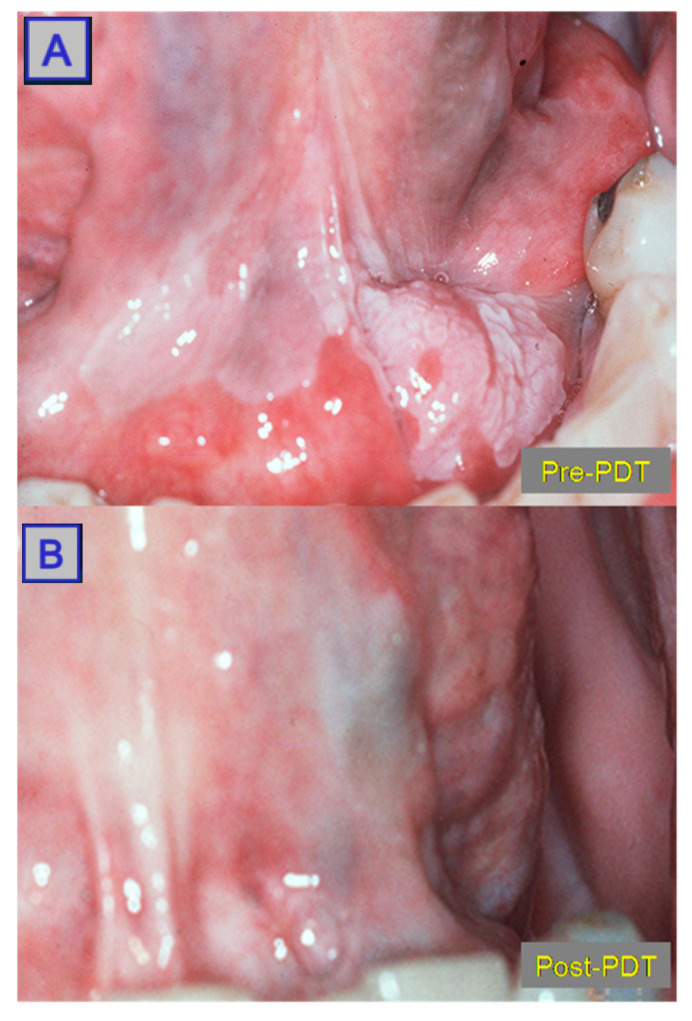
Images showing an oral squamous cell carcinoma pre-PDT (**A**) with superficial tissue changes. Post-PDT (**B**) showing significant tissue regeneration, indicating a positive response to treatment. Reprinted with permission from ref. [36]. Copyright 2011 Lasers Surg. Med.

One of the major benefits of PDT is its ability to be used safely together with all other conventional treatment modalities (Figure 10). As demonstrated by a study conducted by Wang and colleagues, 11 patients with locally advanced OSCCs were treated with platinum-based induction chemotherapy and 5-aminolevulinic acid photodynamic therapy (ALA-PDT), followed by surgery. Of these 11 patients, the response rate was 90.9% (at one-month post-treatment) and there was no local recurrence; one patient was excluded due to an unrelated myocardial infarction. This study, although having a small sample size, demonstrates the safety of ALA-PDT. Of the four patients who experienced serious side effects, these were all chemotherapy related, with mild oedema being reported post-PDT [67].

A recent systematic review and meta-analysis on 18 articles and 900 patients conducted by Lin et al. concluded that PDT was an effective treatment when used as an adjuvant for surgery. No statistically significant differences were noted between the effectiveness of different photosensitisers, lasers used and radiant exposure. This indicates that there is work to be done to maximise the potential of PDT and optimisation of dosing regimens. However, the studies included in the systematic review were often retrospective and lacked important information on patient survival; therefore, it is difficult to effectively determine differences in protocols. To further determine the value of PDT in the treatment of OSCC, randomised controlled trials are needed with a more careful design than those performed in the past [69].

The management of lower-risk tumours was investigated in a study conducted by Jerjes et al., which examined the effectiveness of m-THPC-PDT in a group of 38 patients. Of the 38 patients included in the study, 5-year survival was 84.2%, which is in line with other treatment modalities. This study contributes to the increasing body of evidence affirming the place PDT has in first-line therapy of low-risk OSCCs, with the added benefit of its being readily repeatable (and usage alongside other treatment modalities) in case of recurrence with fewer side effects [36].

Low-risk tumour treatment was also investigated in a study by de Visscher et al., which compared the use of mTHPC-PDT, when treating T1 and T2 lesions, with surgery. Data were collected from 156 patients treated with PDT and 91 patients treated surgically. The study found that although the rate of re-treatment was higher in the mTHPC-PDT-treated group, there was no overall significant difference in survivability between the two groups. However, T2 tumours treated with mTHPC-PDT fared worse than those treated surgically. An investigation into the differences in morbidity and function was suggested to compare these domains between mTHPC-PDT and surgical management. Differences in outcome could be related to the post-treatment investigations into the remaining tissue. These favour surgical biopsy and histological analysis when compared to visual inspection when mTHPC-PDT is carried out [70].

A study carried out by Ikeda et al. investigated the effectiveness of Photofrin-based PDT for T1 and T2 OSCCs and dysplasia. The data was collected from 25 patients who were treated with PDT and followed-up for a period of 2 years. A complete response was seen in 96% of the patients, with a partial response seen in the remaining patient. Recurrence was seen in 3 patients, and either further PDT, or salvage surgery was carried out to treat the recurrent tumours. All patients experienced post-treatment pain and oedema lasting 3–4 weeks, which was treated with opiate and NSAID analgesia. The study also noted that newer photosensitisers such as mTHPC may achieve better results when treating early OSCC [68].

Due to the complex anatomy of the oral cavity, timely and accurate diagnosis of cancers in the region is difficult. The disease course is often asymptomatic, complicating diagnosis further. Oral potentially malignant diseases, which are significantly more likely to transform into cancers, are seldom diagnosed in a timely manner [71].

Photosensitisers are used in PDT and also in photodiagnosis (PD). 5-ALA is a PS which can be administered via various routes. Alekseeva et al. have developed a sublingual method of application which has the benefits of allowing medical and diagnostic procedures to be carried out independent of the tumour location. The glucose in the dose supports active transport of 5-ALA into cells, and avoids the risk of aspiration associated with anaesthesia when administered orally. The development of a sublingual administration route may improve the efficacy of PDT and PD whilst negating side effects associated with other routes, thus improving tolerability [71].

### 2.3. Oropharyngeal Carcinoma

Malignant neoplasms of the oropharynx occur in four main anatomical sites: the posterior wall of the pharynx, the tongue base, the soft palate, and the tonsillar complex. Of the four main locations, the tonsillar complex is the most common site, with up to 80% of cases estimated to occur in this region. Due to the anatomical location, initial visual inspection is difficult, and as a result, many oropharyngeal malignancies are diagnosed late with advanced disease. Histologically, oropharyngeal cancers are overwhelmingly oropharyngeal squamous cell carcinomas (OPSCC), with a small portion of salivary tumours recorded, among others [72]. OPSCCs are more prevalent in south-east Asia, with India, Sri Lanka and Pakistan reporting high rates of incidence [73]. Risk factors for OPSCC have traditionally been alcohol and tobacco consumption, with human papilloma virus (HPV) infection recently being accepted as a significant risk factor; those with HPV-16-related OPSCC can expect to have a better prognosis [74,75].

For OPSCCs, treatment depends on staging and location, but not on HPV status [76]. Surgery is considered the gold-standard treatment for localised tumours in patients who can tolerate it, with transoral surgery observed to reduce the high risk of morbidity which open surgery carries [77]. In more advanced base of tongue and tonsillar complex disease, surgery with mandibulotomy, used in conjunction with chemotherapy and radiotherapy, is the accepted course of treatment [78].

Minimally invasive techniques are needed in the future to preserve function and minimise treatment-related morbidity. PDT offers promise as a primary or additional treatment to conventional therapies, offering a better side-effect profile (Table 3).

A tertiary centre conducted a retrospective study investigating the role of photodynamic therapy in functionally inoperable oral and oropharyngeal carcinoma. A total of 26 patients received photodynamic therapy for OSCC and OPSCC, with a mixture of surface illumination and interstitial PDT using mTHPC. A total of 76.9% of patients experienced a complete response immediately after treatment, 80.8% of patients were found to have new tumour activity after a follow-up of 27 months, and 96.2% of patients had previous treatment, with the majority [14] undergoing ablative surgery and radiotherapy. The author concluded “PDT is a valuable treatment option in selected patients with oral and/or oropharyngeal HNSCC that induces durable local control in an important fraction of treated patients. The technique has an acceptable toxicity profile” [9].

Karakullukcu et al. analyzed the outcomes of 170 patients who were treated for OSCC and OPSCC using mTHPC-PDT. Response was evaluated using the WHO tumour response criteria. The complete response rate was 70.8% post-treatment, and overall disease-free rates at 5 years were 61% for OPSCC. Of note, the site with the greatest response was that of the oral tongue, with 94.4% of tumours experiencing a complete response. The study indicated a need for increased research into tumour response to PDT at different anatomical subsites. The study also pointed to the difficulty of US access to the oropharynx, especially when trying to assess tumour depth, which is an important parameter for selecting PDT candidates [79].

**Figure 11:**
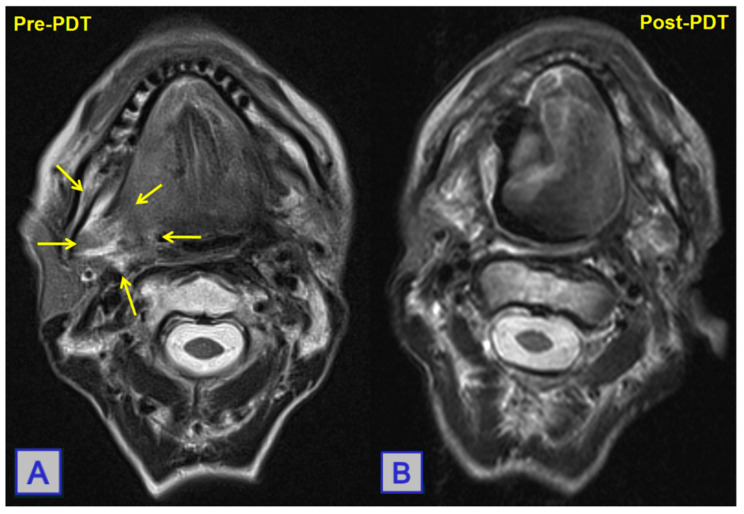
MRI showing a posterolateral tongue-base squamous cell carcinoma. Pre-PDT (**A**), a large mass is seen. Post-PDT (**B**), a reduction in lesion size with widespread tumour necrosis is noted. Reprinted with permission from ref. [10]. Copyright 2010 Clin. Oncol. (R. Coll. Radiol.).

**Figure 12:**
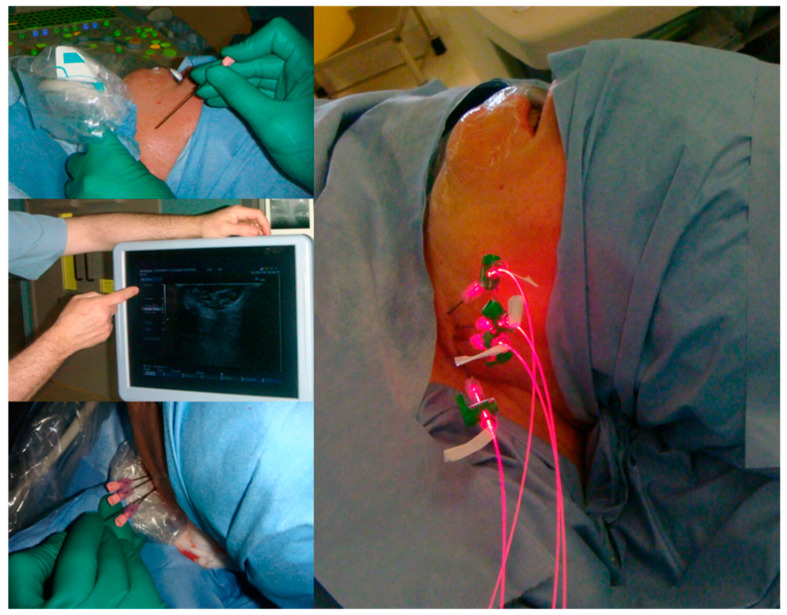
Images of US-guided mapping of needle insertion location in order to avoid the carotid artery. Fibres are introduced through the needles into the tumour bulk, and illumination can be seen. Reprinted with permission from ref. [80]. Copyright 2009 Lasers Surg. Med.

**Figure 13:**
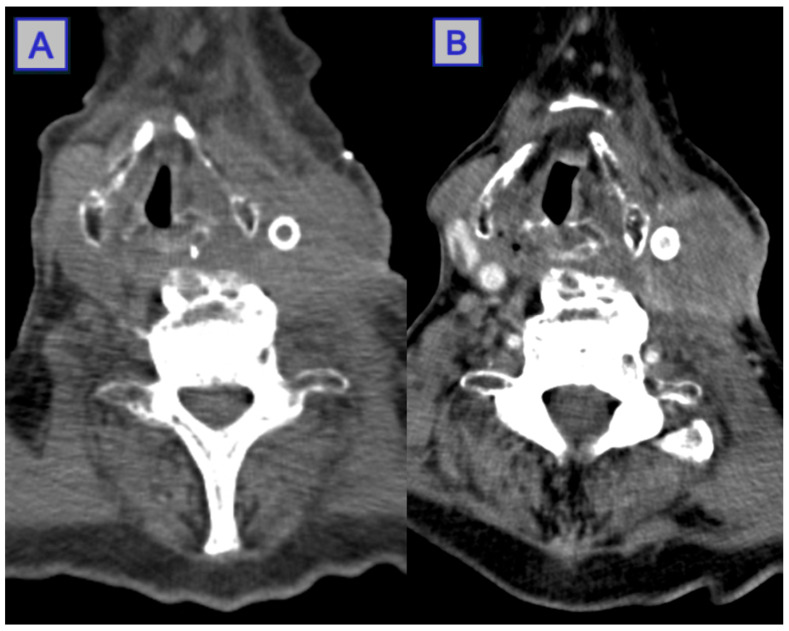
Images of CT scans showing a peri-carotid and left vocal cord lesion causing a shift of the midline. Pre-PDT (**A**), contrast enhanced scan with stent insertion. Post-PDT (**B**), a reduction of midline shift is noted, with evidence of necrotic and inflammatory tissue surrounding the tumour. Reprinted with permission from ref. [81]. Copyright 2010 Photodiagnosis Photodyn. Ther.

The value of PDT in palliation was investigated in a study conducted by Jerjes et al. A total of 21 patients were included in the study, all of whom had been referred for the treatment of recurrent or advanced tongue base cancers, with a mean follow-up of 36 months reported (Figure 11, Figure 12 and Figure 13). Of these patients, two-thirds had not been offered further conventional treatment, and an multi-disciplinary team (MDT) decision was made to offer mTHPC interstitial-PDT (iPDT) under ultrasound guidance. Although survival was measured (eight patients died), the study focused on QoL improvements, and demonstrated improvements in speech and swallowing in over half of the patient group. Over half of the patient group were deemed to be showing a “good response” to treatment, and two patients were found to have a reduction in lesion size. The study concluded that in the group of patients studied, significant improvements were seen, pointing towards the promise of further iPDT use in palliative care [42].

### 2.4. Laryngeal Carcinoma

**Figure 14:**
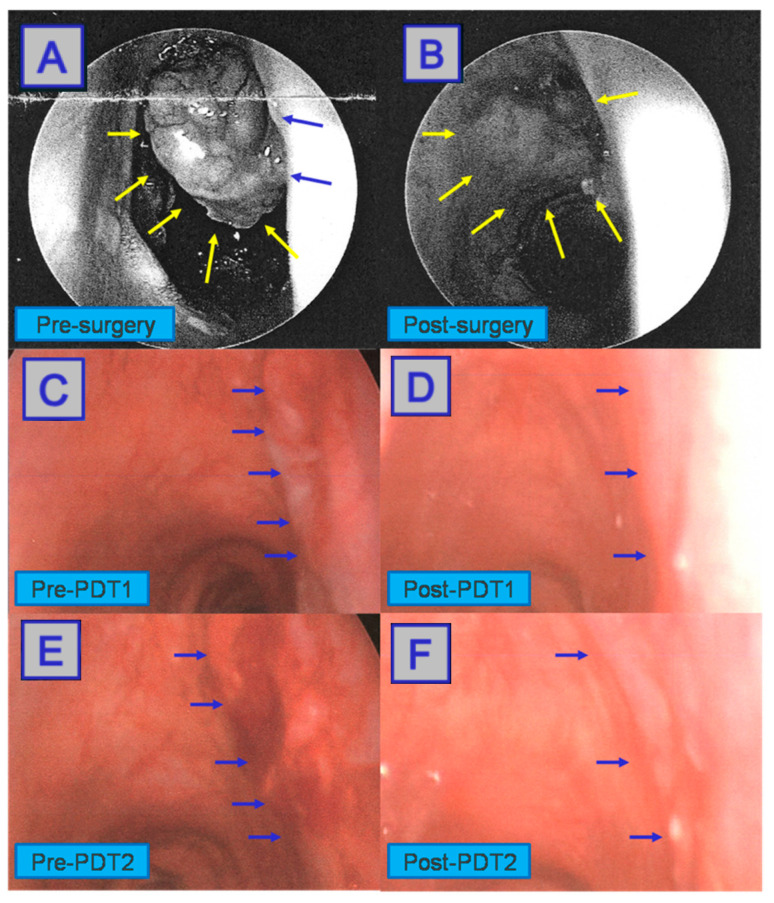
Surgical removal of the initial tumour, shown during intraoperative panendoscopy (**A**) and (**B**). Some tumour regrowth shown (**C**), subsequently treated with PDT. A reduction in regrown tumour bulk is seen (**D**). A second round of PDT carried out on tumour regrowth (**E**), followed by a complete response (**F**). Reprinted with permission from ref. [86]. Copyright 2010 Photodiagnosis Photodyn. Ther.

**Figure 15:**
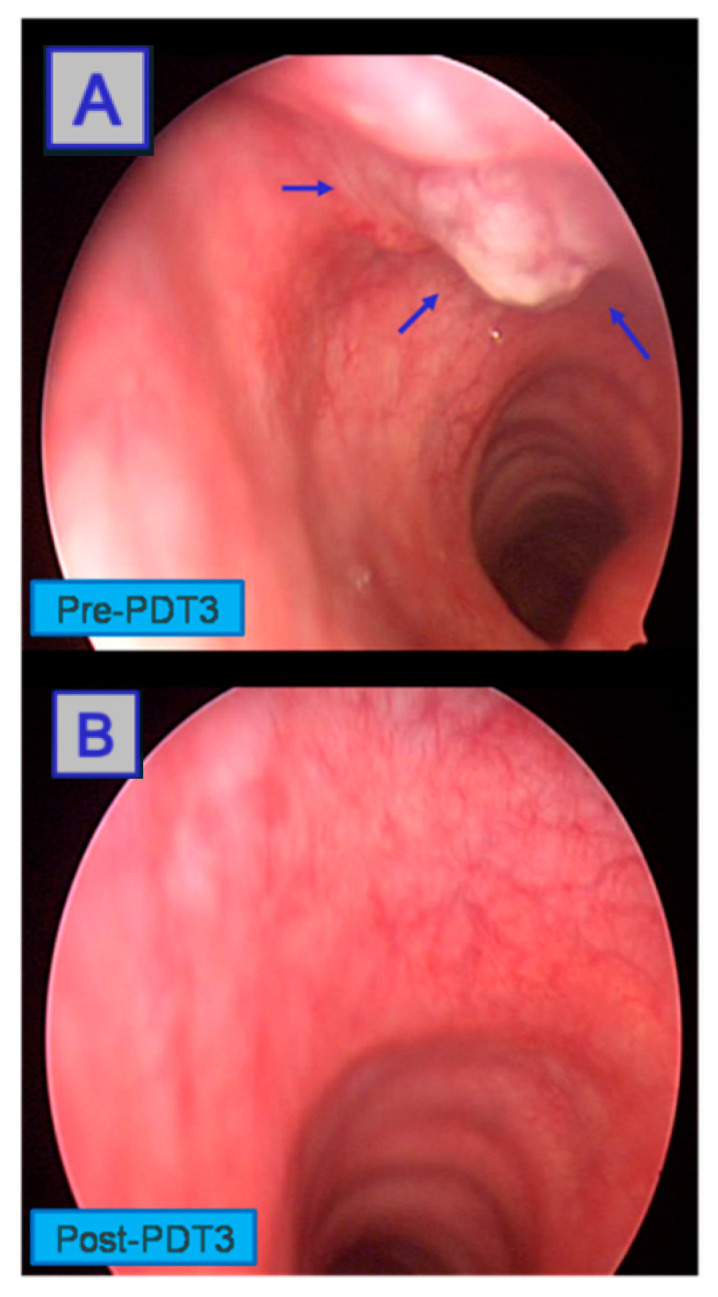
Third round of PDT carried out on remaining tumour regrowth (**A**). Intraoperative panendoscopy shows the complete response to treatment (**B**). Reprinted with permission from ref. [86]. Copyright 2010 Photodiagnosis Photodyn. Ther.

Laryngeal carcinomas constitute one of the most common types of head and neck cancer. Treatment of early laryngeal carcinomas usually involves either surgery or radiotherapy. A single-modality treatment is preferred to reduce the side effects and toxicity, which often include pain, infection and cosmetic defects. Due to the anatomical position of these squamous cell carcinomas, the tumours, or the treatment of them, often cause problems with important functions like swallowing and speech. Therefore, surgical and radiotherapy techniques have evolved to spare organs and restore/preserve function as much as possible. PDT is therefore a useful technique in the management of cancers of this region, because it has little effect on underlying structures [71,72,73,74,75,76,77,78,79,82] (Table 4) (Figure 14 and Figure 15).

Biel treated 115 patients with early laryngeal carcinomas (carcinoma in situ/T1/T2) with photofrin at a dose of 2 mg/kg and DLI of 48 h. A total of 105 patients showed a complete response, at a mean of 91 months follow-up. The remaining 10 were all salvaged (either using further rounds of PDT, surgery or radiotherapy) [44]. Rigual et al. treated six patients with laryngeal lesions: three with laryngeal dysplasia and three with T1 laryngeal carcinoma. A complete response was recorded in five of the patients (all of the dysplasias and two of the T1 tumours), highlighting that PDT is an effective treatment for laryngeal dysplasia and early carcinoma [83].

Von Beckerath et al. published their findings of a descriptive retrospective study discussing cases of laryngeal carcinomas treated using PDT. Between 2000 and 2012, nine patients were treated using PDT, with one patient treated in 1988 also included. The author attributes the relatively low number of patients treated with PDT to the success of conventional treatment modalities in the treatment of such lesions. The patients were treated using different photosensitisers. All seven patients treated with temoporfin had a complete response to PDT. At least half of the patients had an improvement in voice, and none had a worsening. The voice outcome of PDT therefore seems to be comparable with other treatment modalities. Treatment efficacy also seems to be comparable [84].

Shafirstein et al. conducted a study to evaluate the safety profile, dosing and efficacy of 3-(1′-hexyloxyethyl) pyropheophorbide-a (HPPH) PDT for laryngeal cancers, including dysplasias, carcinomas in situ and T1 graded tumours. Whilst earlier studies have shown photofrin^®^ to be efficacious with good preservation of laryngeal function, it leaves patients with persistent skin photosensitisation. In this study, the patients were treated using different light doses, with the maximum tolerated dose (MTD) found to be 100 J/cm^2^. The dose-limiting toxicity was found to be laryngeal oedema, the degree of which was proportional to the dose of light delivered, leading the authors to ponder whether 75 J/cm^2^ would be effective. However, given the small sample sizes in the groups, making judgements on efficacy is of limited value. Nevertheless, this study shows that HPPH is a safe photosensitiser for treatment of laryngeal cancers, providing the light does not exceed 100 J/cm^2^ with 4 mg/m^2^ HPPH [85].

A study by Hosokowa et al. showed an efficacy rate of 100% (PR and CR) for 10 patients with laryngeal cancers. This study also included 23 other cases of head and neck cancer treated with PDT: overall efficacy was 97%, with a CR rate of 72.7% recorded. The authors concluded that PDT is curative for early-stage laryngeal cancer and can preserve laryngeal function [6].

### 2.5. Vascular Anomalies

The term vascular anomalies encompasses a broad variety of lesions related to disordered vascular development. The vast majority are, therefore, congenital in nature. Typically, the lesions are classified into vascular tumours and vascular malformations. The distinction between these groups is based on histopathological assessment of cell turnover: vascular tumours are characterized by abnormal proliferations, whereas vascular malformations do not undergo abnormal cell turnover; instead, these are endothelial-lined channels within vascular tissues. Whilst these are congenital, they grow proportionally with the rest of the body and hence are often missed at birth [87].

Vascular malformations can be further sub-categorised into capillary, venous, lymphatic and arteriovenous malformations. The latter is a fast-flow malformation whilst the former three are described as slow-flow. Capillary malformations (CMs) are often described as “Port Wine Stains” (PWSs). They tend to affect the face, typically the dermatomes of the ophthalmic and maxillary divisions of the trigeminal nerve [87].

**Figure 16:**
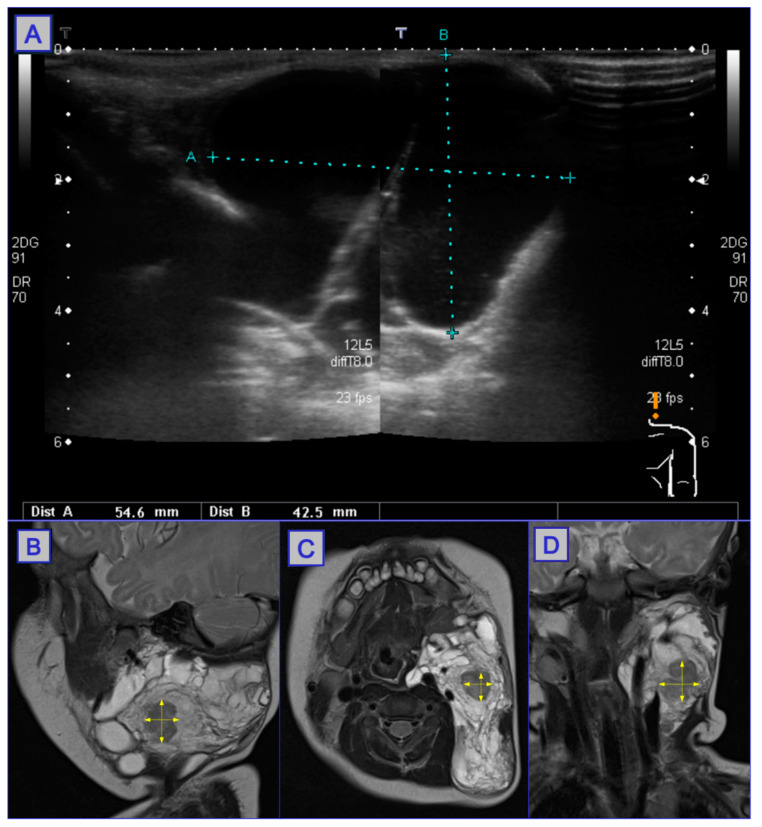
US imaging used to inspect the cystic hygroma (**A**), in which a large cystic lesion can be seen. MRI scanning (**B**–**D**) shows a considerable reduction in lesion size post-PDT. Reprinted with permission from ref. [88]. Copyright 2010 Photodiagnosis Photodyn. Ther.

Jerjes et al. performed ultrasound-guided interstitial photodynamic therapy (US iPDT) on 43 vascular anomalies in the head and neck region (Figure 16). The photosensitiser used was mTHPC at 0.15 mg/kg, with a 96 h DLI reported. MRI imaging was taken 5–6 weeks post-PDT, and the size of the lesions was used as the basis of the radiological assessment of response. A total of 15/43 patients had a significant response (reduction in lesion size by 50–75%), while a further 11 had a moderate response (reduction in lesion size by 25–50%). A total of 12 showed a minimal response (reduction in lesion size by <25%), 4 showed no change with stable disease, and 1 showed progressive disease [35]. Since the sample size was small, and even smaller when considering different types of vascular anomalies, it is hard to draw conclusions. However, this study showed that iPDT may have a role in the treatment of vascular anomalies.

The role of PDT in the treatment of CMs has been studied extensively. Pulsed-dye laser (PDL)-mediated photothermolysis is the current standard treatment for these vascular malformations. Whilst PDL has been shown to be safe and effective, complete-blanching is only achieved in around 10–20%, with around 20% of patients with CM resistant to PDL. The colour of CMs ranges from pale pink to red to purple [89].

In 2011, Zhao et al. demonstrated the efficacy of the photosensitiser hemoporfin (haematoporphyrin monomethyl ether (HMME)) in the treatment of CMs. A total of 39 patients were taken and assigned to either group A or B (laser exposure of 20 or 30 min, respectively) but no statistical difference in efficacy was found between the two groups. Taken therefore as one group, subjective grading of responses 8 weeks post-PDT using the PWS fading method revealed 36 patients showed “good” or “excellent” responses when graded by the investigators: this fell to 35 when graded by the patients themselves. The primary efficacy evaluation was based on standardized photos of the lesions, with fading > 60% deemed as a “significant response”, and between 20% and 60% deemed a “response”. Based on these assessments, 26 patients had a significant response and 8 had a response [90].

A double-blinded, randomised study in 2018 investigated the optimal dose of hemoporfin. A total of 100 patients were taken and split 2:2:1 into low-dose (2.5 mg/kg hemoporfin), high-dose (5 mg/kg hemoporfin) and control (placebo) groups. After 8 weeks, the 20 patients who received the placebo were split equally between low- and high-dose regimes. No statistically significant differences in response between the control group and low-dose group were found. However, the high-dose group did respond statistically significantly better than the other two groups. The secondary efficacy evaluation was subjective grading by patients and investigators, as in the 2011 trial. Subjective assessment found that the high-dose patients reported the best satisfaction, whilst the low-dose group did report better satisfaction than the control group [91]. Whilst the efficacy of the high-dose regimen was better than the low-dose, there was a statistically significantly higher rate of adverse effects in the high-dose groups. However, all adverse effects were well tolerated, and there were no significant adverse effects reported in either group.

A 2013 study by Gao et al. of 15 patients with CMs showed PDT (using hemoporfin as the photosensitiser) to be as effective and tolerable as PDL, the current-standard treatment. CMs were shown to respond well to PDT, with blanching rates after a single-session superior to single-session PDL [89]. Zhao et al. conducted a large randomised control trial at 8 hospitals in China, involving 440 patients. The trial used hemoporfin 5 mg/kg as the photosensitiser. At 8 weeks post-treatment, the hemoporfin group achieved significantly higher rates of at least some improvement (defined as at least a 20% decrease in the size of the lesion) of 89.7%, compared to 24.5% for the placebo group [92]. This trial adds to increasing evidence that PDT is an effective treatment for CMs and should be considered as an alternative therapy for those resistant to the conventional PDL. See Table 5.

## 3. Conclusions

PDT has been shown to be as effective as conventional therapies for the treatment of oral, oropharyngeal, nasopharyngeal and laryngeal cancers, as well as vascular anomalies in the head and neck region. Since the light source is targeted onto the area of the lesion, and the activated photosensitiser acts over an extremely small radius, the effects of PDT are largely confined only to diseased tissue. PDT also causes minimal damage to underlying structures, further confining the damage to healthy tissue.

Photosensitisers accumulate in all cells, and as a result, there is a period of photosensitivity after the therapy which effectively confines the patient indoors to avoid sunlight exposure. This period is a serious disruption to the patient’s work and social lives. Further, due to the specific wavelengths at which current photosensitisers are excited at, the depth of lesions which can be treated with PDT is limited. This restricts the number of eligible lesions.

Most of the studies in this review focused on cases of recurrent or persistent cancers resistant to conventional treatments. Whilst these cases have shown PDT to be safe and effective, future applications to investigate could include primary treatment of head and neck cancers. Additionally, the development of new generations of photosensitisers could reduce the duration of residual photosensitivity and increase the depth of lesions that can be treated using PDT, improving tolerability and expanding the number of eligible lesions, respectively.

## Figures and Tables

**Table 1:** Summary of recent clinical trials investigating the use of Photodynamic therapy(PDT) in nasopharyngeal carcinoma (NPC).

Study and Year of Publication	Number of Patients	Photo-Sensitiser	Photosensitiser Dose	Results at the End of Follow Up (Mean Follow-Up Time)
Abbas et al., 2012 [39]	7 (2 stage III, 5 stage IV)	m-THPC	0.15 mg/kg, drug-light interval 96 h	5 died (3 tumour related), 2 alive (36 months)
Nyst et al., 2012 [58]	22	m-THPC	*n* = 8: 0.15 mg/kg, drug-light interval 96 h *n* = 7: 0.10 mg/kg, drug-light interval 48 h *n* = 7 0.075 mg/kg, drug-light interval 24 h	7 out of 8 patients who received the recommended dose survived (37.8 months) (1 died of pneumonia 2 days post-PDT). 6 out of 7 who received 0.1 mg/kg PS died (3 related to disease, 1 of unknown cause and 2 unrelated to disease). 3 out of 7 receiving 0.075 mg/kg PS died (all due to disease). Overall survival 55% at 3 years 100% complete response *.
Succo et al., 2014 [59]	6	m-THPC	0.15 mg/kg, drug-light interval 96 h	2 died (disease-related), 1 alive with disease, 3 disease-free (24–71 months of follow-up).
Stoker et al., 2015 [60]	21	m-THPC	0.15 mg/kg, drug-light interval 96 h	10 died, 2 alive with disease and 9 alive and disease-free (32 months).

* Only 17/22 patients had treatment response evaluated.

**Table 2:** Summary of recent clinical trials evaluating the efficacy of PDT in the treatment of oral squamous cell carcinoma.

Study	Number of Patients	Photosensitiser	Dose	Response (At the End of Follow-Up)		
				CR	PR	NR
Wang et al., 2021 [67]	11	5-aminolevulinic acid (5-ALA)	10% cream	7	3	1
Jerjes et al., 2011 [36]	38	Meso-tetrahydroxyphenyl chlorin (mTHPC)	0.15 mg/kg	26		
Ikeda et al., 2013 [68]	25	Photofrin^®^	2 mg/kg	24	1	0

**Table 3:** Summary of recent studies investigating the efficacy of PDT in the treatment of oropharyngeal carcinomas.

Study	Number of Patients	Photosensitiser	Dose	Response (At the End of Follow-Up) *		
				CR	PR	NR
Lambert et al., 2021 [9]	26	mTHPC	0.15 mg/kg	20	3	3
Karakullukcu et al., 2011 [79]	170	mTHPC	0.15 mg/kg	119	36	15
Jerjes et al., 2011 [42]	21	mTHPC	0.15 mg/kg	2	13	6

* Complete response defined as no visual or biopsy-proven evidence of disease; Partial response defined as a reduction in maximum diameter of affected area by 50% or more; No change defined as a reduction in maximum diameter of affected area by less than 50%.

**Table 4:** Summary of recent studies where PDT was given to patients with laryngeal cancer.

Study	Number of Patients	Photosensitiser	Dose	Response (At the End of Follow-Up) *		
				CR	PR	NR
Biel [44]	115	Photofrin ^®^	2 mg/kg	105	10	0
Rigual et al. [83]	6	Photofrin ^®^	2 mg/kg	5	0	1
Von Beckerath et al. [84]	10	Photofrin ^®^ (*n* = 2), temoporfin (*n* = 7), both (*n* = 1)	Photofrin 2 mg/kg or 5 mg/kg Temoporfin 0.15 mg/kg	7	3	0
Shafirstein et al. [85]	29 (30 lesions)	HPPH	4 mg/m^2^	20	6	4 **
Hosokawa et al. [6]	10	Photofrin ^®^	2 mg/kg	9	1	0

CR = complete response; HPPH = 3-(1′-hexyloxyethyl) pyropheophorbide-a; PR = partial response; NR = no response. * Complete response defined as no visual or biopsy-proven evidence of disease; Partial response defined as a reduction in maximum diameter of affected area by 50% or more; No change defined as a reduction in maximum diameter of affected area by less than 50%. ** includes one case of progressive disease.

**Table 5:** Summary of recent clinical trials evaluating the efficacy of PDT in the treatment of vascular anomalies.

Study and Year of Publication	Number of Patients	Photo-Sensitiser	Results
Jerjes et al., 2011 [37]	43	m-THPC	Radiological assessment (MRI) taken 5–6 weeks post-PDT showed 15 significant responses, 11 moderate responses, 12 minimal responses, 4 no change with stable disease, 1 progressive disease **.
Zhao et al., 2011 [90] *	39	Hemoporfin	26 patients showed significant response; 8 showed response to PDT treatment ***
Gao et al., 2013 [89] *	15	Hemoporfin	9 red-coloured CMs showed 11–24% blanching rate after a single session of PDL compared to 22–55% for PDT. 6 purple-coloured CMs showed 8–33% improvement after PDL compared to 30–45% for PDT.
Zhao et al., 2016 [92] *	440	Hemoporfin	89.7% of the PDT group showed at least some improvement, compared to 24.5% of the placebo group.
Wu et al., 2018 [91] *	100	Hemoporfin	Primary efficacy assessment revealed that for the high-dose group, 30/40 patients (75%) showed at least some improvement, with 16/40 (40%) showing at least great improvement. This compares with 16 (40%) and 1 (2.5%) out of 40 for the low dose group, respectively. In the control group, 3/20 patients (15%) showed some improvement, with 17/20 (85%) showing no improvement.

* study only evaluated capillary malformations. ** Significant response defined as reduction in lesion size by 50–75%; moderate response: a reduction of 25–50%; minimal response: a reduction of <25%. *** significant response defined as fading between 20% and 60% based on standardised photos; response defined as <20% fading.

## Data Availability

Not applicable.
